# Asiaticoside inhibits TGF-β1-induced mesothelial-mesenchymal transition and oxidative stress via the Nrf2/HO-1 signaling pathway in the human peritoneal mesothelial cell line HMrSV5

**DOI:** 10.1186/s11658-020-00226-9

**Published:** 2020-05-29

**Authors:** Junyi Zhao, Jun Shi, Yun Shan, Manshu Yu, Xiaolin Zhu, Yilin Zhu, Li Liu, Meixiao Sheng

**Affiliations:** Renal Division, Affiliated Hospital of Nanjing University of Chinese Medicine, 155 Hanzhong Road, Nanjing, 210029 Jiangsu Province China

**Keywords:** Human peritoneal mesothelial cells (HPMCs), Mesothelial-mesenchymal transition (MMT), Reactive oxygen species (ROS), TGF-β/Smad, Nrf-2/HO-1

## Abstract

**Background:**

Peritoneal fibrosis (PF) is a frequent complication caused by peritoneal dialysis (PD). Peritoneal mesothelial cells (PMCs), the first barrier of the peritoneum, play an important role in maintaining structure and function in the peritoneum during PD. Mesothelial-mesenchymal transition (MMT) and oxidative stress of PMCs are two key processes of PF.

**Purpose:**

To elucidate the efficacy and possible mechanism of asiaticoside inhibition of MMT and ROS generation in TGF-β1-induced PF in human peritoneal mesothelial cells (HPMCs).

**Methods:**

MMT and ROS generation of HPMCs were induced by TGF-β1. To explain the anti-MMT and antioxidant role of asiaticoside, varied doses of asiaticoside, oxygen radical scavenger (NAC), TGF-β receptor kinase inhibitor (LY2109761) and Nrf2 inhibitor (ML385) were used separately. Immunoblots were used to detect the expression of signaling associated proteins. DCFH-DA was used to detect the generation of ROS. Transwell migration assay and wound healing assay were used to verify the capacity of asiaticoside to inhibit MMT. Immunofluorescence assay was performed to observe the subcellular translocation of Nrf2 and expression of HO-1.

**Results:**

Asiaticoside inhibited TGF-β1-induced MMT and suppressed Smad signaling in a dose-dependent manner. Migration and invasion activities of HPMCs were decreased by asiaticoside. Asiaticoside decreased TGF-β1-induced ROS, especially in a high dose (150 μM) for 6 h. Furthermore, ML385 partly abolished the inhibitory effect of asiaticoside on MMT, ROS and p-Smad2/3.

**Conclusions:**

Asiaticoside inhibited the TGF-β1-induced MMT and ROS via Nrf2 activation, thus protecting the peritoneal membrane and preventing PF.

## Introduction

Peritoneal fibrosis (PD) is one of the effective treatments for patients with end-stage renal disease (ESRD), whose efficacy depends on the structure and function of the peritoneum. Compared with hemodialysis (HD), PD has the advantage of retention of residual renal function (RRF), leading to a higher quality of life [1]. HD and PD treatment have the same mortality and their impact on survival does not seem to change over time [2]. The PMC monolayer is the first barrier against external injury factors, maintaining the peritoneum integrity, functional stability and damage repair [3]. Under physiological conditions, PMCs secrete various cytokines to perform immune surveillance and regulate inflammation and tissue repair, maintaining the structure and function of the peritoneum. Unfortunately, accumulated evidence has highlighted that long-term exposure to conventional PD solutions (PDS) may damage morphology and function of PMCs, leading to progressive PF and dialysis failure [4].

MMT has been widely considered as an early and crucial process in PF [5]. Despite recent controversies about the source of myofibroblasts [6, 7], MMT is still a potential therapeutic target for the prevention of PF [8]. Oxidative stress is a disordered state between oxidative molecules and insufficient antioxidant defense, causing tissue injury and systemic damage. Currently, a growing number of studies have found that PDS-induced ROS may alter peritoneal structure and function during long-term PD, whereas antioxidants may prevent such changes [9]. It has been reported that several signaling pathways, including oxidative stress, TGF-β/Smad, non-Smad and noncoding RNAs, participate in regulating the process of MMT [10]. Myofibroblasts are known to provide an unfavorable environment for generation of pro-fibrotic cytokines and ROS. These results suggest that MMT and ROS generation are important causes of PF in PD. Patients with these complications ultimately quit PD after several years of therapy.

To clarify the role of asiaticoside in inhibiting TGF-β1-induced MMT and ROS generation, we focused on the pro-fibrotic signaling pathway TGF-β/Smad and the antioxidant signaling pathway Nrf2/HO-1 to illustrate a potential method for inhibiting PF. TGF-β1 promotes fibrosis mainly by activating TGF-β type I and type II receptors, and then activating Smad protein to mediate MMT [11]. The transcription factor nuclear factor erythroid-2-related factor 2 (Nrf2) could regulate the induction of genes encoding antioxidant proteins and phase2 detoxifying enzymes by activating antioxidant response elements (AREs). In addition, heme oxygenase 1 (HO-1) responds to oxidative stress and is up-regulated by Nrf2, exerting antioxidant properties [12]. Several recent studies have shown that Nrf2 alleviates kidney damage by inducing antioxidant enzymes in vivo and in vitro [13]. Despite a deep understanding of the mechanism of PF, there is no effective treatment for this. Therefore, effective antifibrotic therapies remain to be explored. To date, several studies have focused on herbal medicine as alternative treatments.

**Fig. 1:**
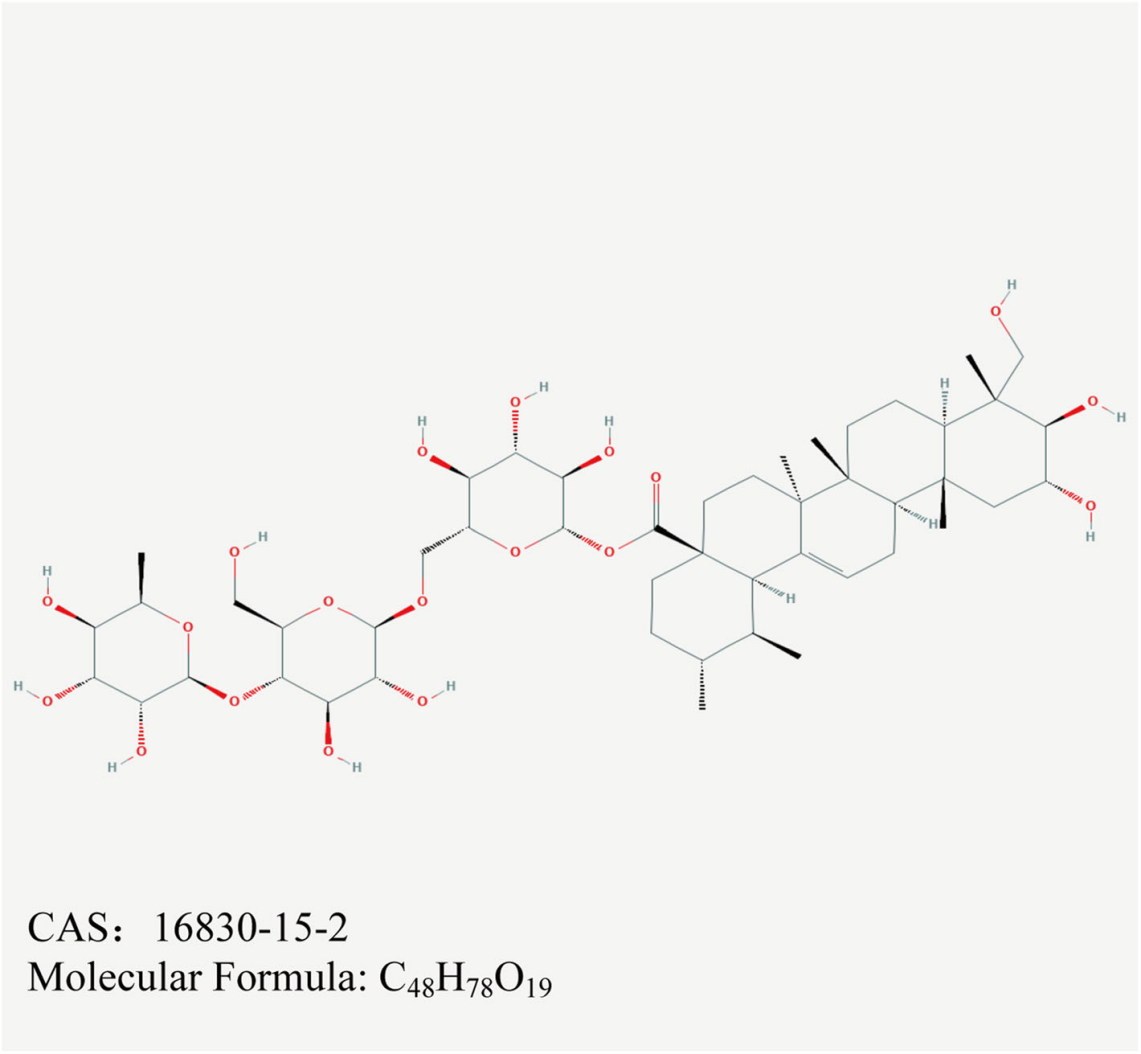
Chemical structure of asiaticoside. (Abbreviated AS in the figures)

*Centella asiatica* (L.) Urban (Apiaceae) has been used in traditional Chinese medicine in treating various diseases for over 2000 years. Asiaticoside (shown in Fig.1) is the main component of triterpenoid saponins extracted from *Centella asiatica* with a clear formula. Emerging evidence has indicated that asiaticoside shows antioxidant, anti-inflammatory, anti-fibrotic and other pharmacological effects [14–16]. In the PD field, the protective effects of asiaticoside against MMT and PD-related ROS remain unknown. In this study, we used TGF-β1-induced HPMCs to investigate the role of asiaticoside in MMT and ROS generation and to elucidate its underlying mechanisms.

## Materials and methods

### Cell lines and culture conditions

HMrSV5 cells (Lian Mai Bioengineering Co., Ltd., Shanghai, China) are immortal cell lines and are equivalent to HPMCs isolated from human peritoneum. HPMCs were cultured in 1640 basic medium (RPMI 1640; Gibco, USA) supplemented with 1% penicillin-streptomycin (Invitrogen; Carlsbad, CA, USA) and 10% fetal calf serum (FCS; Invitrogen) in a humidified incubator with 5% CO2 at 37 °C. All experiments were carried out after cells were seeded in culture plates containing 1% FCS for 24 h. 10 ng/cm^3^ TGF-β1 (R&D; Minneapolis, MN, USA) was used to induce MMT and ROS in HPMCs. Asiaticoside (C_48_H_78_O_19_; CAS: 16830–15-2; HPLC ≥98%; Yuanye Biotechnology Co., Ltd. Shanghai, China) was dissolved in DMSO for a stock concentration of 1.5 × 10^5^ μM. The final concentration of DMSO in the medium was lower than 0.1% to avoid affecting the cell viability.

### Cell viability assay

Cell Counting Kit-8 (CCK-8; Dojindo, Kumamoto, Japan) was used to measure cell viability. Cells were seeded at a density of 2 × 10^3^ cells per well in 96-well plates and subjected to various interventions. Then CCK-8 solution (10 mm^3^) was added to each well, incubating for another 1 h at 37 °C. Optical density was measured at 450 nm (Bio-Rad 550, USA).

### Immunoblotting assay

Cells were lysed in ice-cold RIPA lysis buffer (Thermo Fisher Scientific, Waltham, MA, USA) containing 0.1 mM PMSF. The lysates were centrifuged, and the supernatants were collected for immunoblotting. NE-PER nuclear and cytoplasmic extraction reagents (Thermo) were used to obtain nuclear and cytoplasmic proteins, respectively. The protein concentration was measured using the BCA Protein Assay Kit (Thermo). Extracted protein lysates were separated at a quality of 20 μg/lane using SDS-PAGE and electro transferred onto PVDF membranes. After blocking with 5% BSA in TBST, the membranes were incubated with primary antibody at 4 °C overnight, followed by incubation with HRP-conjugated anti-mouse/rabbit IgG secondary antibodies for 1 h. Finally, the bands were soaked with immobilon ECL ultra western HRP substrate (Millipore, Bedford, USA) and visualized using a chemidoc imaging system. The following antibodies were used: E-Cadherin (3195), Vimentin (5741), α-SMA (19245), p-Smad2/3 (8828), Smad2/3 (8685), β-actin (4970), H3 (4499) and secondary HRP-conjugated anti-rabbit (7074) antibodies were obtained from Cell Signaling Technology (Boston, MA, USA). HO-1 (sc136960) and secondary HRP-conjugated anti-mouse (sc516102) antibodies were obtained from Santa Cruz Biotechnology. Nrf2 (ab62352) was obtained from Abcam (Cambridge, UK). TGF-β1 receptor blocker LY2109761 was obtained from APExBIO Technology (Houston, USA). Nrf2 inhibitor ML385 was obtained from Selleck Chemicals (Houston, TX, USA).

### Transwell migration assay

The transwell chamber was placed in a 24-well plate, and HPMC cells at the logarithmic phase (treated with 150 μM asiaticoside with or without 10 ng/cm^3^ TGF-β1 for 24 h) were selected, which were then digested and centrifuged, and the cells were diluted with serum-free RPMI 1640 medium to 2.5 × 10^5^/cm^3^. 200 mm^3^ of cell suspension was added to each upper transwell chamber (pore size = 8 μm), and 800 mm^3^ of 1640 basic medium was added to each well of the lower chamber. After 24 h of culture, the chamber was removed, and the upper layer was wiped off with a cotton swab. Migrated cells were fixed with 4% paraformaldehyde for 15 min and stained with 0.1% crystal violet for 15 min. Five microscopic fields were selected randomly to take pictures and calculate the number of migrated cells.

### Wound healing assay

The wound healing assay was used to detect cell migratory ability. HPMCs (1.0 × 10^5^/cm^3^) were seeded in 24-well plates. After cells grew to 90–100% confluence, a wound line was produced with a sterile pipette tip based on a ruler. Cellular debris was removed by washing with PBS, and cells were allowed to migrate for 24 h after intervention. Images were taken at 0 and 24 h after wounding under an Olympus BX45 inverted microscope. The relative distance traveled by the leading edge from 0 to 24 h was detected using ImageJ software (*n* = 5).

### Measurement of ROS levels

The generation of ROS was detected using 2′,7′-dichloroflfluorescin diacetate (DCFH-DA; Sigma, Saint Louis, MO, USA). After treatment, cells were incubated with 30 μM DCFH-DA in a dark environment at 37 °C for 30 min. After three washes with PBS, each well was fixed with 1 cm^3^ formaldehyde for 30 min at room temperature. After three washes with PBS, the cells were analyzed using a fluorescence microscope (Carl Zeiss, Oberkochen, Germany). Cells with green fluorescence were considered as ROS-positive ones (at least 50 cells from a single capture field).

### Immunofluorescence assay

Cells (5 × 10^4^/well) were grown to adherence on 8-well glass Nunc Lab-Tek chamber slides and were treated with TGF-β1 with or without asiaticoside. After treatment for 24 h, the slides were washed to fix, permeate, and block. Next, cells were incubated with primary antibody at 4 °C overnight, and then incubated with fluorescent secondary antibody for 1 h at room temperature, and stained with DAPI (Beyotime Biotechnology Co., Ltd. Shanghai, China) for 15 min. Images were acquired with a Zeiss AX10 fluorescence microscope.

### Statistical analysis

We performed at least 3 individual experiments for each object on different days. All data were expressed as the mean ± standard error of the mean (SEM) using the SPSS 19.0 statistical software. For those data with few samples, we performed logarithmic transformation to make it conform closely to a normal distribution. Comparisons of two populations were performed by Student′s t-test. For multiple comparisons, one-way analysis of variance (ANOVA) followed by Dunnett′s test was employed. Values of P less than 0.05 were considered statistically significant.

## Results

### Asiaticoside showed no effect on cell growth and apoptosis under 150 μM

**Fig. 2:**
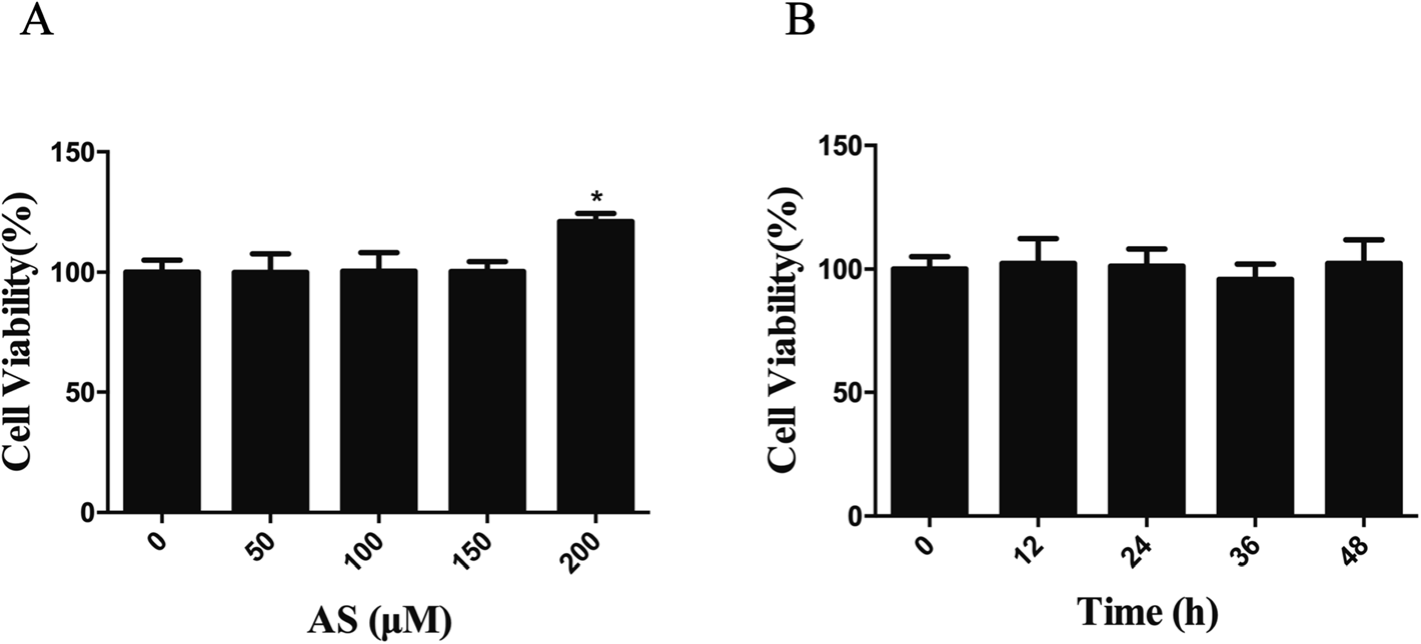
Effect of asiaticoside on HPMCs viability. **Notes:** Cell viability was evaluated using CCK-8 assay. Data were expressed as the percentages of living cells versus the control. **(A)** HPMCs were treated with various dose of asiaticoside (0, 50, 100, 150 and 200 μM) for 24 h or **(B)** with 150 μM of asiaticoside for various times (0, 12, 24, 36 and 48 h). **Abbreviations:** HPMCs, human peritoneal mesothelial cells; CCK-8, cell counting kit-8

According to the results from the CCK-8 assay, neither enhanced cell proliferation nor apoptosis was found when the concentration was less than 150 μM or 150 μM for various times, but cell proliferation was enhanced when the concentration of asiaticoside was above 200 μM (Fig. 2a-b). Accordingly, 150 μM of asiaticoside was the dose chosen for the subsequent experiments.

### Asiaticoside attenuated TGF-β1-induced MMT by inhibiting TGF-β/Smad signaling pathway

**Fig. 3:**
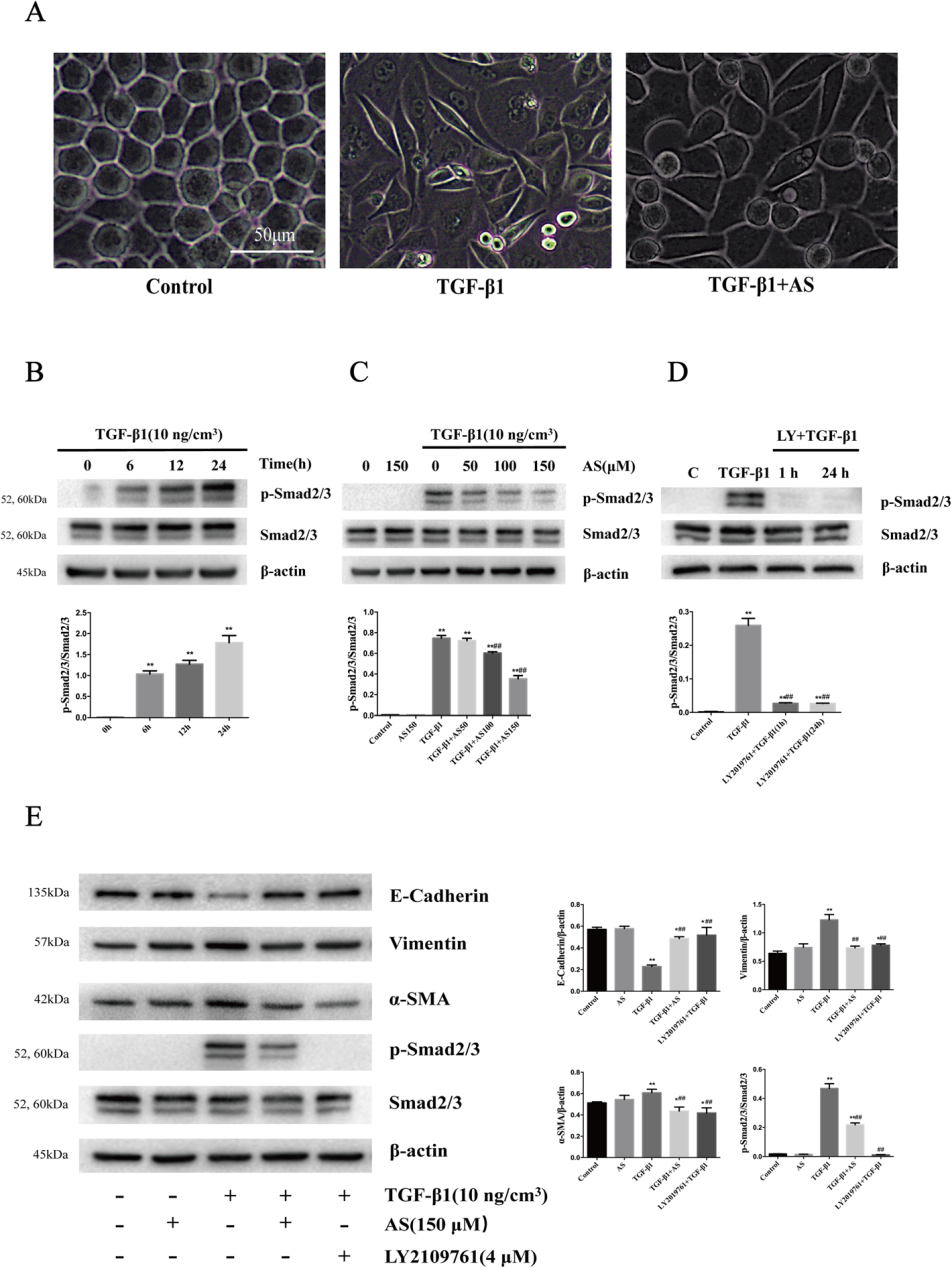
Effect of asiaticoside on TGF-β1-induced activation of Smad2/3 and MMT in HPMCs. **Notes: (A)** After treatment with TGF-β1 (10 ng/cm^3^) or/and asiaticoside (150 μM), the morphologic alterations of HPMCs were observed using a microscope (scale bar = 50 μm). **(B)** HPMCs were treated with TGF-β1 (10 ng/cm^3^) for various times (0, 6, 12, 24 h) and subjected to immunoblot for Smad-related proteins. **(C)** HPMCs were treated with asiaticoside at various concentrations (0, 50, 100, 150 μM) with or without TGF-β1 (10 ng/cm^3^) treatment for 24 h. The expression of Smad2/3 and p-Smad2/3 was detected by immunoblotting. **(D)** Immunoblot showed relative levels of Smad2/3 phosphorylation at 1 h and 24 h post-treatment with LY2109761. **(E)** HPMCs were divided into a vehicle group, an asiaticoside group (treated with 150 μM asiaticoside), a TGF-β1 group (treated with 10 ng/ cm^3^ TGF-β1), a TGF-β1 + asiaticoside group (treated with 150 μM asiaticoside + 10 ng/cm^3^ TGF-β1) and a LY2109761 + TGF-β1 group (pretreated with LY2109761 at 4 μM 1 h prior to 10 ng/cm^3^ TGF-β1). After incubation for 24 h, immunoblot was performed to detect relative proteins. β-actin was used as a loading control. The densitometric analysis of the expression of MMT markers, Smad2/3 and p-Smad2/3 are shown as unique figures. Data are expressed as mean ± SEM, *p < 0.05 vs. control; **p < 0.01 vs. control; #p < 0.05 vs. TGF-β1 treatment; ##p < 0.01 vs. TGF-β1 treatment. **Abbreviations:** AS, asiaticoside; TGF-β1, transforming growth factor-β1; LY, TGF-β receptor kinase inhibitor (LY2109761); p-Smad2/3, phosphorylated Smad2/3

**Fig. 4:**
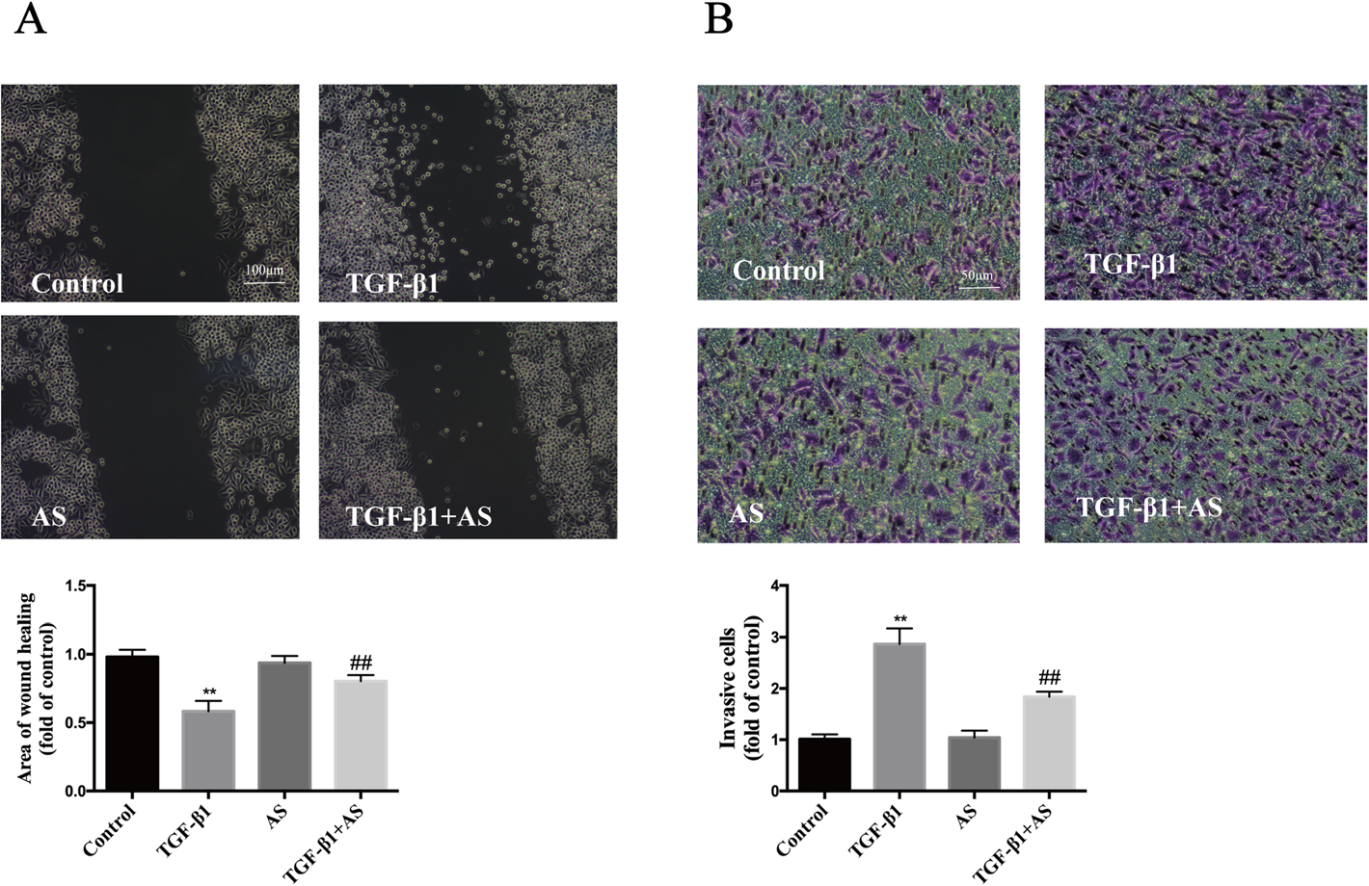
Effect of asiaticoside on TGF-β1-induced migration ability of HPMCs. **Notes:** HPMCs were exposed to asiaticoside (150 μM) with or without TGF-β1 (10 ng/cm^3^) and subjected to microscope to visualize the lateral **(A)** and longitudinal **(B)** migration of cells. Quantitative measurements of the fluorescence intensities were conducted using ImageJ software. Five random fields for each insert were counted, and three independent experiments were performed in each group. *n* = 3. **Abbreviations:** AS, asiaticoside; TGF-β1, transforming growth factor-β1

After treatment with 10 ng/cm^3^ TGF-β1 for 24 h, HPMCs appeared elongated and branched, with a loss of paving stone-like appearance, whereas asiaticoside treatment reduced these changes from a cuboidal shape to elongated spindle-shaped cells (Fig. 3a). Subsequently, we observed the effect of 10 ng/cm^3^ TGF-β1 on time gradients. The expression of p-Smad2/3 was strongly increased at 24 h after treatment (Fig. 3b). Asiaticoside attenuated the p-Smad2/3 expression in TGF-β1-stimulated HPMCs in a dose-dependent manner (Fig. 3c). Immunoblot analysis demonstrated that the expression of the mesothelial cell marker (E-Cadherin) decreased and the expressions of mesenchymal cell markers (Vimentin and α-SMA) increased under TGF-β1 stimulation. Asiaticoside alleviated TGF-β1-induced expressions of Vimentin and α-SMA, with an increase in the expression of E-Cadherin. These results were accompanied by enhanced phosphorylated Smad2/3 (p-Smad2/3), a Smad signaling pathway activated marker protein, suggesting that Smad2/3 was activated in TGF-β1-induced MMT. Furthermore, after pretreatment with the TGF-β receptor kinase inhibitor LY2109761, both asiaticoside and LY2109761 attenuated MMT and the Smad signaling pathway, indicating that asiaticoside has similar effects as TGF-β inhibitor (Fig. 3d-e). In addition, the migratory ability of HPMCs was enhanced under the TGF-β1 (10 ng/cm^3^) condition, whereas the addition of asiaticoside reduced the migration of HPMCs. These results were further confirmed by transwell migration assay and wound healing assay (Fig. 4a-b). These observations suggested that the anti-MMT effect of asiaticoside may be due to the downregulation of Smad signaling in TGF-β1-stimulated HPMCs.

### Asiaticoside inhibited ROS production independently of TGF-β/Smad signaling

**Fig. 5:**
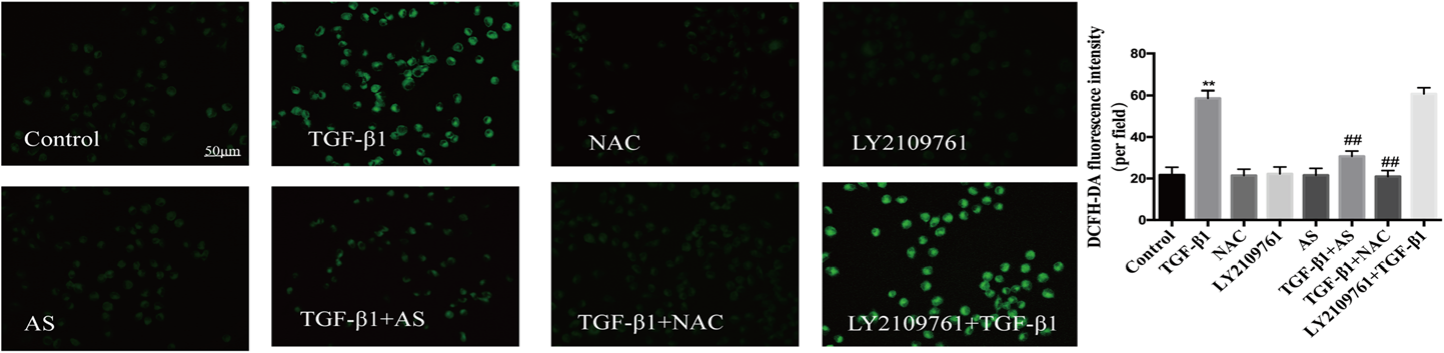
Effect of asiaticoside on reactive oxygen species (ROS) production in TGF-β1-treated HPMCs. **Notes:** HPMCs were divided into a vehicle group, an asiaticoside group (treated with 150 μM asiaticoside), a TGF-β1 group (treated with 10 ng/cm^3^ TGF-β1), a TGF-β1 + asiaticoside group (treated with 150 μM asiaticoside + 10 ng/cm^3^ TGF-β1), an NAC group (treated with 5 mM NAC), an NAC + TGF-β1 group (pretreated with NAC at 5 mM 2 h prior to 10 ng/cm^3^ TGF-β1), a LY2109761 group (treated with 4 μM LY2109761), and a LY2109761 + TGF-β1 group (pretreated with LY2109761 at 4 μM 1 h prior to 10 ng/cm^3^ TGF-β1). After incubation for 24 h, DCFH-DA fluorescence in cultured cells was analyzed by fluorescence microscopy (scale bar = 50 μm). Quantitative measurements of the fluorescence intensities were conducted using ImageJ software. **Abbreviations:** AS, asiaticoside; TGF-β1, transforming growth factor-β1; DCFH-DA, 2′,7′-dichlorodihydrofluorescein diacetate; NAC, n-acetylcysteine; LY2109761, TGF-β receptor kinase inhibitor

ROS levels were markedly increased after treatment with 10 ng/cm^3^ TGF-β1 for 24 h. Both asiaticoside and NAC (oxygen radical scavenger, 5 mM) attenuated this increase in TGF-β1-exposed HPMCs, which indicated the antioxidant effect of asiaticoside. In addition, LY2109761 treatment did not affect the TGF-β1-induced ROS production in HPMCs (Fig.5). These results suggested that asiaticoside-mediated attenuation of ROS is independent of TGF-β/Smad signaling pathway.

### Nrf2 was activated by asiaticoside in HPMCs

**Fig. 6:**
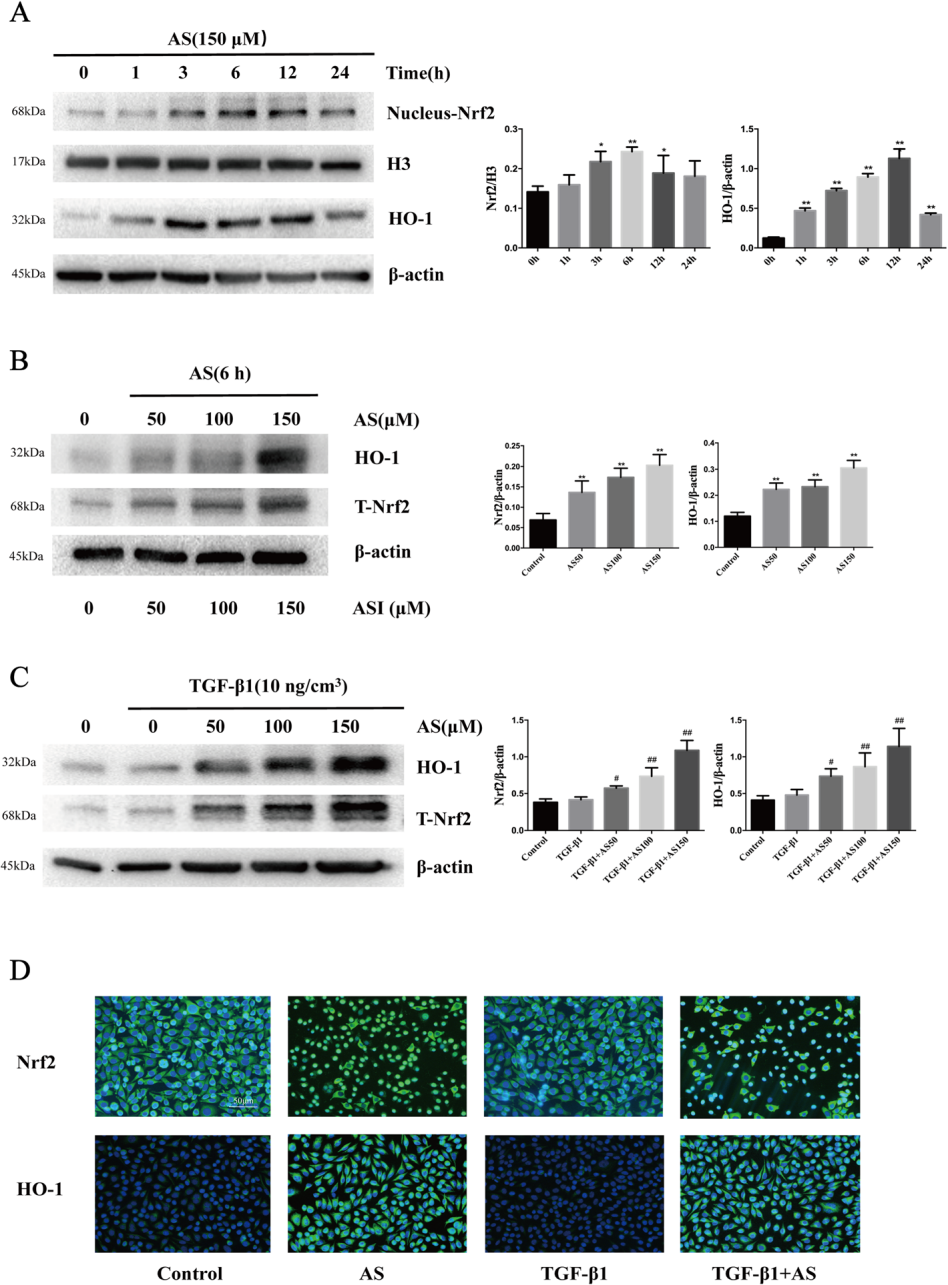
Effect of asiaticoside on Nrf2 activation in HPMCs. **Notes: (A)** After asiaticoside (150 μM) treatment for various times (0, 1, 3, 6, 12, 24 h), the expression of Nrf2 in the nucleus and HO-1 expression were detected by immunoblotting. Histone H3 and β-actin were used as the loading controls for the nucleus and cytosol, respectively. **(B)** After treatment with various doses of asiaticoside (0, 50, 100, 150 μM) for 6 h, Nrf2 and HO-1 expression were detected by immunoblotting. **(C)** After treated with asiaticoside at various concentrations (0, 50, 100, 150 μM) and TGF-β1 (10 ng/cm^3^) treatment for 6 h, Nrf2 and HO-1 expression were detected by immunoblotting. β-actin was used as a loading control. Densitometric analysis of the expression of Nrf2 and HO-1 are shown as unique figures. Data are expressed as the mean ± SEM, *p < 0.05 vs. control, **p < 0.01 vs. control; #p < 0.05 vs. TGF-β1 treatment; ##p < 0.01 vs. TGF-β1 treatment. **(D)** Immunofluorescence assays were performed to observe the subcellular translocation of Nrf2. Nrf2 expression in the membrane, cytoplasm and nucleus were visualized based on the green fluorescent signal obtained by fluorescence microscopy (scale bar = 50 μm). **Abbreviations:** AS, asiaticoside; TGF-β1, transforming growth factor-β1; Nrf2, nuclear factor erythroid-2-related factor 2; HO-1, heme oxygenase 1

Previous studies have reported that this traditional Chinese medicine plant *Centella asiatica* has antioxidant effects resulting from the activation of Nrf2 and the expression of its downstream antioxidant enzymes in different disease areas. To examine the optimal moment for asiaticoside to activate Nrf2, we treated HPMCs with asiaticoside (150 μM) at different times. Nuclear Nrf2 (nNrf2) levels markedly increased after treatment with asiaticoside for 3–6 h. However, the nuclear translocation of Nrf2 was reduced 12 h after asiaticoside treatment. Consistent with this, asiaticoside increased the expression of HO-1, which is a well-studied Nrf2 target gene (Fig. 6a). After treatment with various doses of asiaticoside (50–150 μM) for 6 h, both total Nrf2 (T-Nrf2) and HO-1 levels were significantly increased in a dose-dependent manner (Fig. 6b). These results suggest that Nrf2 could be activated by different concentrations of asiaticoside at 6 h. Accordingly, asiaticoside intervention of 150 μM concentration for 6 h was chosen for the subsequent experiments. After treatment with 10 ng/cm^3^ TGF-β1 for 24 h and intervention with 150 μM asiaticoside for 6 h, T-Nrf2 and HO-1 levels in TGF-β1-stimulated HPMCs were both increased in dose-dependent manners (Fig. 6c). Immunofluorescence assay further confirmed the phenomenon that asiaticoside promoted Nrf2 to enter the nucleus and enhanced the expression of the downstream antioxidant enzyme HO-1 (Fig. 6d). All the above results suggested that asiaticoside activated the Nrf2/HO-1 signaling pathway, which may exert an antioxidant effect in inhibiting TGF-β1-induced ROS.

### Nrf2 played a key role in the inhibitory effect of asiaticoside on TGF-β1-induced MMT and ROS production

**Fig. 7:**
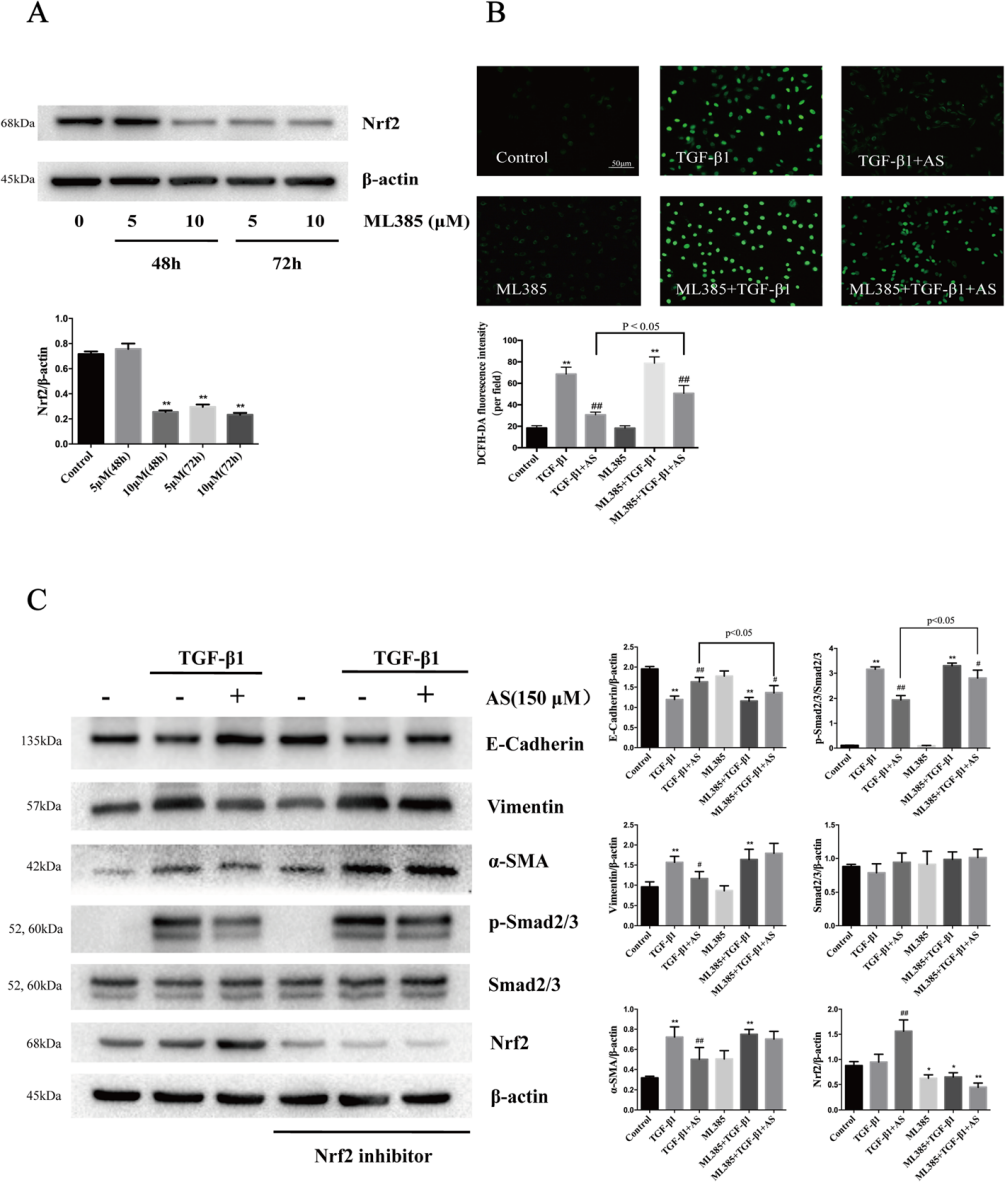
The role of Nrf2 activation in the inhibitory effect of asiaticoside on TGF-β1-induced MMT and ROS production in HPMCs. **Notes: (A)** After HPMCs treatment with two concentration of ML385 (5, 10 μM) and subsequently incubation for 48 h and 72 h, Nrf2 was analyzed by immunoblotting. Data are shown as the mean ± SEM, *p < 0.05 vs. control, **p < 0.01 vs. Control. **(B)** HPMCs were divided into a vehicle group, a TGF-β1 group (treated with 10 ng/ml TGF-β1), a TGF-β1 + asiaticoside group (treated with 150 μM asiaticoside + 10 ng/cm^3^ TGF-β1), a ML385 group (treated with 10 μM ML385 for 72 h), a ML385 + TGF-β1 group (pretreated with ML385 at 10 μM 72 h prior to 10 ng/cm^3^ TGF-β1), and a ML385 + TGF-β1 + asiaticoside group (pretreated with ML385 at 10 μM 72 h prior to 150 μM asiaticoside + 10 ng/cm^3^ TGF-β1). After incubation for 24 h, DCFH-DA fluorescence in cultured cells was analyzed by fluorescence microscopy (scale bar = 50 μm). Quantitative measurements of the fluorescence intensities were conducted using ImageJ software. **(C)** Immunoblot was performed to detect MMT markers, Smad-related proteins and Nrf2 expression. Data are shown as the mean ± SEM, *p < 0.05 vs. control, **p < 0.01 vs. control; #p < 0.05 vs. TGF-β1 treatment; ##p < 0.01 vs. TGF-β1 treatment. **Abbreviations:** AS, asiaticoside; TGF-β1, transforming growth factor-β1; Nrf2, nuclear factor erythroid-2-related factor 2; p-Smad2/3, phosphorylated Smad2/3; ML385, Nrf2 inhibitor; ROS, reactive oxygen species

To verify the role of asiaticoside-mediated Nrf2 activation in HPMCs, we measured TGF-β1-mediated ROS generation after treatment with Nrf2 inhibitor (ML385) in HPMCs. Although asiaticoside still inhibited TGF-β1-induced ROS production after treatment with ML385, the inhibitory effect was partially reversed. However, compared with Nrf2 inhibited cells, the inhibitory effect of TGF-β1 plus asiaticoside treatment was still significant (Fig. 7a-b). In addition, it was verified by immunoblotting that inhibition by Nrf2 partially alleviated the inhibition effect of asiaticoside on MMT and p-Smad2/3 (Fig. 7c). These results suggested that asiaticoside had antioxidant effects against TGF-β1-induced ROS production through the Nrf2/HO-1 pathway, leading to the inhibition of fibrogenic gene expression.

## Discussion

**Fig. 8:**
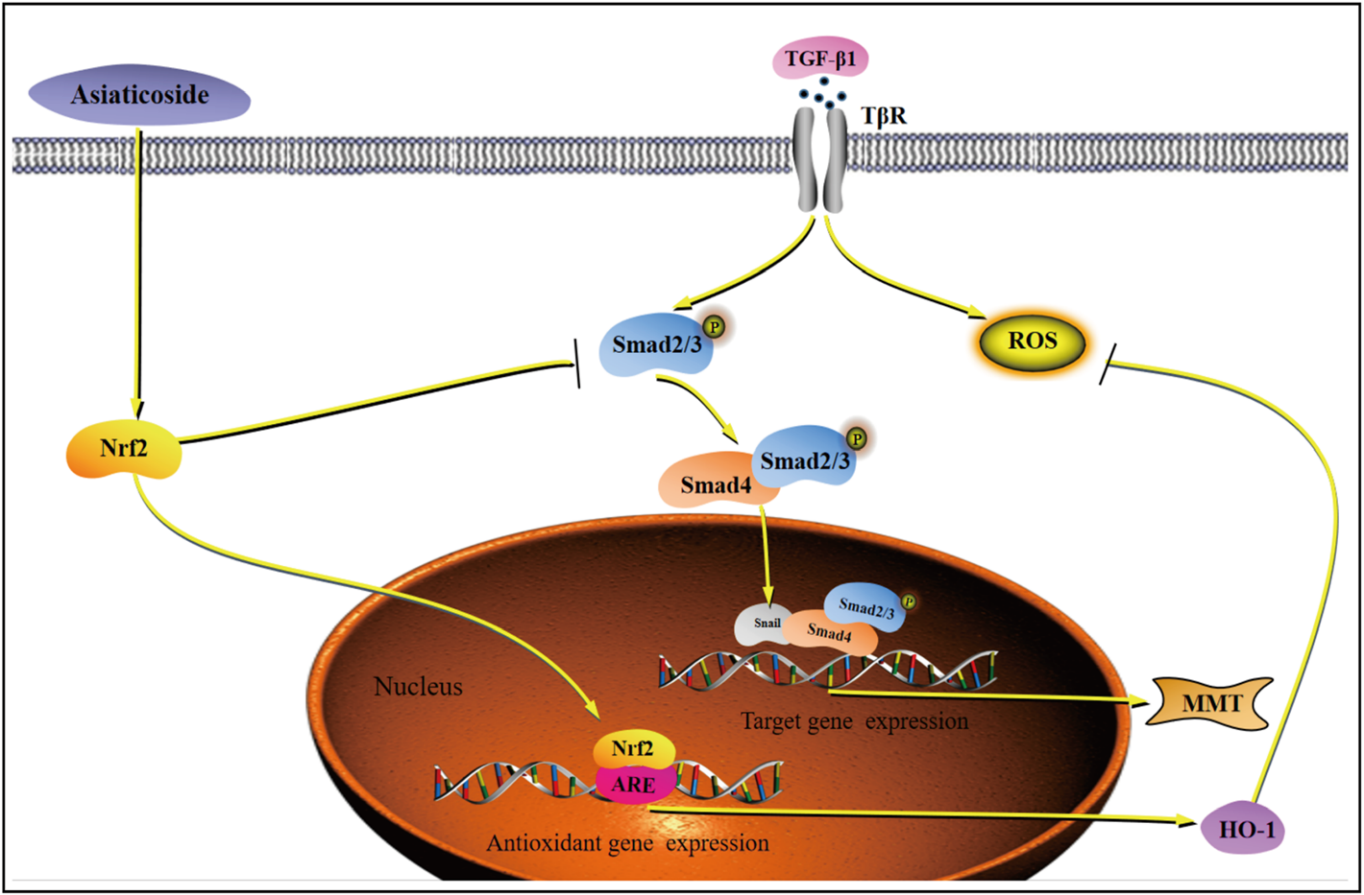
Schematic diagram illustrating the mechanism by which asiaticoside inhibited MMT and ROS generation

In this study, it was found that (i) asiaticoside inhibits MMT and ROS generation in TGF-β1-treated HPMCs; (ii) the beneficial effect of asiaticoside on MMT is attributable to inhibition of TGF-β/Smad signaling, which has no effect on ROS; (iii) asiaticoside inhibited the TGF-β1-induced oxidative stress and Smad signaling via Nrf2 activation, thus preserving peritoneal membrane function and preventing PF (Fig. 8).

MMT is an important cellular process strongly associated with PF observed in long-term PD patients [17]. Pathological changes of PMCs include exfoliation of mesothelial cells and thickening of the submesothelial layer, which is characterized by increased myofibroblasts, collagen deposition, and new blood vessels. PMCs acquire a mesenchymal phenotype, and directly participate in the pathogenesis of PF. Therefore, prevention and/or reversal of the regulation of MMT can be an effective method for treating PF. TGF-β1 plays a central role in MMT of HPMCs [18]. Many TGF-β1 blockers are used for anti-MMT action in vivo and *vitro*, such as TGF-β1 receptor blocker and neutralizing antibody. In the last years, many authors have focused on the beneficial effects of natural herbs or their active ingredients on maintaining peritoneal integrity [19, 20]. But none of them have been used clinically, and the safety is unknown.

The most important finding of this study is validation of the anti-MMT effect of asiaticoside in HPMCs. It is already known that TGF-β1 forms a complex with the TGF-β type II receptor, and then the activated TGF-β type I receptor phosphorylates the downstream Smad2/3 proteins. Next, phosphorylated Smad2/3 and Smad4 combine to transmit signaling to the nucleus, where they work in concert with transcription factors such as Snail and Twist to suppress the expression of mesothelial cell markers and to activate the expression of mesenchymal cell markers [21]. Starting from this evidence, our study found that TGF-β1 induced MMT with activation of Smad2/3 by phosphorylation in HPMCs. We confirmed activation of the TGF-β/Smad signaling pathway and MMT assessed by immunoblotting, which was blocked by asiaticoside and LY2109761. Furthermore, transwell and wound-healing assays illustrated that asiaticoside treatment reduced transmigration in TGF-β1-treated cells to a certain extent. All the above results confirmed that asiaticoside can inhibit MMT via TGF-β/Smad signaling. Earlier studies have revealed the anti-MMT effect of asiaticoside in keloid fibroblasts and lung epithelial cells [14]. However, there have been no reports in the context of PD. This study is the first one to demonstrate the regulatory effect of asiaticoside on PMCs MMT by Smad signaling.

It is generally accepted that oxidative stress is associated with the occurrence and development of PF in PD patients, which is related to RRF. The composition of the PD solution (low pH, high glucose, elevated osmolality, advanced glycation end products and glucose degradation products) is responsible for the accumulation of oxidation products. Therefore, oxidative stress has been considered as an important factor in PF [22]. Asiaticoside has been reported as an antioxidant agent that ameliorates cell apoptosis and alleviates fibrotic change by inhibiting ROS production. Under the stimulation of TGF-β1, ROS generation was significantly increased in HPMCs. Similar to the effect of NAC, asiaticoside eliminated the overproduction of ROS. Accumulated studies have demonstrated that TGF-β1 stimulated the production of ROS in various cell types, which in turn activated TGF-β and mediated many fibrogenic effects. Some studies have shown that TGF-β/Smad signaling plays a vital role in oxidative stress [23]. We investigated the relationship between TGF-β/Smad signaling and oxidative stress in PMCs; however, LY2109761 treatment did not decrease ROS production, indicating that TGF-β1-induced ROS in HPMCs does not depend on TGF-β/Smad signaling. While TGF-β1-induced Smad signaling is a critical event in the progression of oxidative stress, the role of non-Smad mechanisms in the overproduction of ROS is still critical [24, 25]. These results revealed asiaticoside to be an antioxidant agent through non-Smad pathways, and a multi-target compound with anti-MMT and anti-ROS mechanisms.

In order to further study the specific mechanism and antioxidant target of asiaticoside, we clarified the transduction of Nrf2/HO-1 signaling. Nrf2 is a redox-sensitive transcription factor which provides an antioxidant defense mechanism by regulating the induction of genes encoding antioxidant proteins and phase2 detoxifying enzymes through the activation of AREs [26]. Previous studies have reported that activation of Nrf2 and its downstream gene HO-1 plays an antioxidant role in many cell lines [12]. In the last years, many researchers have focused on the antioxidant effects of natural products and their extracts [27–29]. In our study, interestingly, both nNrf2 and HO-1 levels increased rapidly after asiaticoside treatment for 3–6 h. After treatment with various doses of asiaticoside (50–150 μM) for 6 h, both T-Nrf2 and HO-1 levels were significantly increased. These results confirmed that asiaticoside acts as an Nrf2 activator in a dose-dependent manner. After treatment with 10 ng/cm^3^ TGF-β1 for 24 h, asiaticoside intervention for 6 h was still able to increase the expressions of T-Nrf2 and HO-1 in a dose-dependent manner. Immunofluorescence assay further confirmed that asiaticoside promoted Nrf2 nuclear translocation. These results indicated that asiaticoside served an antioxidant role via activating Nrf2/HO-1 signaling in HPMCs.

Furthermore, a specific role of Nrf2 in asiaticoside-mediated suppression of ROS was verified using the Nrf2/HO-1 inhibitor ML385. ML385 can directly interact with Nrf2 protein and binds to the Neh1 binding region of Nrf2, thus preventing binding of the Nrf2-MAFG complex to the promoter ARE sequence and reducing the transcriptional activity. After treatment with ML385, asiaticoside’s effect on TGF-β1-induced ROS generation was partly ameliorated. These results confirmed that TGF-β1-induced ROS generation in HPMCs was dependent on Nrf2 activation. To our knowledge, few data have demonstrated the activation of Nrf2/HO-1 signaling inhibiting ROS production and MMT in HPMCs. However, it was limited to phenomenon observation and lacked mechanism research and efficacy verification [30]. In our study, we illustrated for the first time that asiaticoside can inhibit ROS production by targeting the Nrf2/HO-1 signaling pathway in the PD field. Previous studies have found that Nrf2 activation inhibits TGF-β/Smad signaling and MMT in tissue fibrosis [31]. Adenovirus-mediated overexpression of Nrf2 prevented MMT and ROS generation in TGF-β1-treated liver and renal cells, which was accompanied by decreased expression of p-Smad2/3. In contrast, Nrf2 knockdown by siRNA prevented this alteration [32, 33]. To verify whether asiaticoside can inhibit MMT and TGF-β/Smad signaling pathways by activating Nrf2, we applied ML385 pretreatment and the experimental results showed that pretreatment with Nrf2 inhibitor partly reversed the inhibitory effect of asiaticoside on the TGF-β1-stimulated MMT and p-Smad2/3 expression. These data indicated that Nrf2 is a key target working in concert with asiaticoside on TGF-β/Smad signaling and oxidative stress, leading to the inhibition of MMT and ROS generation.

To our knowledge, asiaticoside is a large molecule and the studies so far have provided no evidence about a specific receptor or potential binding site. Although it seems difficult to enter the cytosol, we have good reason to believe that asiaticoside indeed interacts with cytoplasmic proteins according to the database. The associations between asiaticoside and relative cytoplasmic/nuclear proteins have been described in many articles (21 studies for TNF-α, 13 for IL-6 and 7 for Smad2). Furthermore, anti-inflammatory activities in LPS-stimulated mouse RAW264.7 cells were assessed as the nitrite level at 1 to 100 μM after 24 h by the Griess method [34]. It is noted that asiaticoside (*Centella asiatica* extract) is more often used in dermatology, while our study demonstrates for the first time its protection of human peritoneum. With the progression of pharmaceutical research, more specific targets/analogs/functional structure are wanted to elucidate the properties of the new drug.

Taken together, our results suggest that asiaticoside effectively suppresses TGF-β1-induced MMT and ROS generation via Nrf2 activation in HPMCs. This result indicates a potential therapeutic effect of asiaticoside on PF.

## Data Availability

All data from this study are available in this published article.

## Abbreviations

AS: asiaticoside
CCK-8: cell counting kit-8
DAPI: 4′,6-diamidino-2-phenylindole
DCFH-DA: 2′,7′-dichlorodihydrofluorescein diacetate
DMSO: dimethyl sulfoxide
ESRD: end-stage renal disease
HD: hemodialysis
HO-1: heme oxygenase 1
HPMCs: human peritoneal mesothelial cells
LY: TGF-β receptor kinase inhibitor (LY2109761)
ML385: Nrf2 inhibitor
MMT: mesothelial-mesenchymal transition
NAC: n-acetylcysteine
Nrf2: nuclear factor erythroid-2-related factor 2
PBS: phosphate buffer saline
PD: peritoneal dialysis
PDS: peritoneal dialysis solution
PF: peritoneal fibrosis
PMCs: peritoneal mesothelial cells
p-Smad2/3: phosphorylated Smad2/3
ROS: reactive oxygen species
RRF: residual renal function
TGF-β1: transforming growth factor-β1
